# Modelling fertility in rural South Africa with combined nonlinear parametric and semi-parametric methods

**DOI:** 10.1186/s12982-018-0073-y

**Published:** 2018-03-02

**Authors:** Robert W. Eyre, Thomas House, F. Xavier Gómez-Olivé, Frances E. Griffiths

**Affiliations:** 1 Centre for Complexity Science, University of Warwick, Coventry, CV4 7AL UK; 2 School of Mathematics, University of Manchester, Manchester, M13 9PL UK; 3 Medical Research Council/Wits University Rural Public Health and Health Transitions Research Unit (Agincourt), School of Public Health, Faculty of Health Sciences, University of the Witwatersrand, Johannesburg, South Africa; 4 Warwick Medical School, University of Warwick, Coventry, CV4 7AL UK; 5 Centre for Health Policy, University of the Witwatersrand, Johannesburg, South Africa

**Keywords:** Fertility, Age-pattern, Socio-economic status pattern, Agincourt, Nonlinear model, Parametric model, Semi-parametric model, Gaussian process regression

## Abstract

**Background:**

Central to the study of populations, and therefore to the analysis of the development of countries undergoing major transitions, is the calculation of fertility patterns and their dependence on different variables such as age, education, and socio-economic status. Most epidemiological research on these matters rely on the often unjustified assumption of (generalised) linearity, or alternatively makes a parametric assumption (e.g. for age-patterns).

**Methods:**

We consider nonlinearity of fertility in the covariates by combining an established nonlinear parametric model for fertility over age with nonlinear modelling of fertility over other covariates. For the latter, we use the semi-parametric method of Gaussian process regression which is a popular methodology in many fields including machine learning, computer science, and systems biology. We applied the method to data from the Agincourt Health and Socio-Demographic Surveillance System, annual census rounds performed on a poor rural region of South Africa since 1992, to analyse fertility patterns over age and socio-economic status.

**Results:**

We capture a previously established age-pattern of fertility, whilst being able to more robustly model the relationship between fertility and socio-economic status without unjustified a priori assumptions of linearity. Peak fertility over age is shown to be increasing over time, as well as for adolescents but not for those later in life for whom fertility is generally decreasing over time.

**Conclusions:**

Combining Gaussian process regression with nonlinear parametric modelling of fertility over age allowed for the incorporation of further covariates into the analysis without needing to assume a linear relationship. This enabled us to provide further insights into the fertility patterns of the Agincourt study area, in particular the interaction between age and socio-economic status.

## Background

The measurement of fertility rates and their relationships to socioeconomic variables are essential to the analysis of the population dynamics of that society. For South Africa, whose history of Apartheid has resulted in a very socio-economically diverse population, the ability to examine trends and patterns in fertility is even more important when trying to assess the development of the country. In the last few decades the country has experienced a number of health and demographic shifts including the HIV pandemic, the rise in prevalence of noncommunicable disease [1], and the decline over time of fertility itself [2]. The calculation of fertility rates from various data sources across the country and sub-Saharan Africa as a whole has proven useful in looking at the impact of HIV/AIDS [3, 4], increased education [5], delayed marriage [4], premarital reproduction [4, 6, 7], contraceptive use [4], and the development of refugee populations [8], as well as more administrative issues such as the evaluation of potentially unreliable Apartheid-era data [9].

Most of this research, as is typical in epidemiology, has relied on established statistical analysis methods of parametric and generalised linear regression, despite more recent innovation in statistical analysis in recent years. Fertility rates are often only examined empirically, leaving the conclusions drawn vulnerable to noise that could exist within the data [10–15]. Linear and logistic regression techniques are commonly used, but are very constraining in their assumption of a linear relationship between fertility and (transforms of) the various covariates considered [3, 7, 16]. Often there is no reason to believe these relationships to be linear at all. A variety of nonlinear models for fertility over age have been developed, such as the Hadwiger, Gamma, and Beta functions [6, 17]. However these models fail to incorporate further covariates in anything more than a linear fashion [2], and also impose their own strong assumptions (although these are potentially much better justified than generalised linearity).

Here we present more general methods for examining the relationship between fertility and various covariates, focusing on age and socio-economic status, by combining a standard nonlinear parametric model of fertility rates over age with the use of Gaussian process regression to bring in further covariates that we do not have well-established models for. In using a parametric model over age, we make sure to capture the nonlinear relationship shown to exist between fertility and age in other work [6, 17]. Gaussian process regression, which produces a distribution of nonlinear functions of fertility over the covariates of interest, then allows us to find nonlinear relationships between fertility and these other covariates without having to define a precise parametric form to the relationships that would force possibly unfounded assumptions onto the results. We then apply this method to data from the Agincourt health and socio-demographic surveillance system (HDSS), an annual census round performed on residents of villages in a poor rural region of South Africa since 1992 [18].

## Methods

### Data

For our analysis we used data from the Agincourt Health and Socio-Demographic Surveillance System (HDSS), run by the Medical Research Council/University of the Witwatersrand Rural Public Health and Health Transitions (Agincourt) Research Unit. Details on its methodology have been published elsewhere [18, 19].

In brief, the Agincourt HDSS is an annual update round of the baseline census performed in 1992. In each round demographic data is collected including births, deaths, and migration. Health information is collected at regular bases and since 2006 a new system allows the linkage of census data with morbidity data at the existing Primary Health System in the study area. Originally it covered 57,600 people in 8900 households in 20 villages [19], and by 2011 it had increased to 90,000 people in 16,000 households in 27 villages [18]. The area is characterised by high unemployment, poor quality education, and poor quality land that makes agricultural farming difficult.

We created a database out of the Agincourt HDSS selecting women who were living in a household in the HDSS dataset during the years that socio-economic status was collected (2001, 2003, 2005, 2007, 2009, and 2011). The inclusion criteria for each year were individuals of all ages who had a recorded date of birth and no date of death proceeding the selected year, and belonged to a household that supplied enough information to calculate an absolute socio-economic status (SES) index. The total sample size was 224,643, where an observation was defined as a woman in an individual year who meets all inclusion criteria. Some women were counted as multiple observations due to appearing in the census dataset in multiple years. Though we did consider the inclusion of other covariates such as education (measured in number of years of education achieved), our analysis focused on fertility (defined as the fraction of women associated with each set of covariate values who experienced a live birth) over age (measured in years) and SES (measured by Agincourt’s household absolute SES index, which averages a set of quantitative measures of the amounts of different types of assets the household possesses [20]), both of which we measured at the midyear point for each year.

In order to calculate sensible values for the empirical fertility rates so that it could be used as the dependent variable of a regression, we binned the observations to set covariate values by splitting them into quantiles and then setting their covariate values to the midpoints for the quantiles they belong to. The precise number of quantiles used for binning each covariate was chosen by a combination of cross-validation and goodness-of-fit techniques, more detail of which is given in the description of the model below. In the end, the preferred quantiles were 125-quantiles for age and 25-quantiles for SES.

Examples of sample sizes and average fertility rates in our chosen dataset for various age and SES ranges in each year are shown in Tables 1 and 2.

**Table 1:** Sample sizes for different ranges of age (in years) and socio-economic status for each year, given to aid comparison of the analytical results to the data

Age	SES	Years					
		2001	2003	2005	2007	2009	2011
10–20	1–2	3063	2883	1772	1033	828	788
	2–3	5333	5784	6497	6402	8097	6769
	3–4	370	362	356	651	1156	1819
20–30	1–2	2046	1915	1183	716	578	570
	2–3	3718	4291	5214	5223	6980	6305
	3–4	254	241	279	567	1125	1832
30–40	1–2	1537	1331	827	502	445	409
	2–3	2644	2960	3439	3427	4448	3842
	3–4	187	210	178	401	678	1104
40–50	1–2	990	911	558	337	278	275
	2–3	1631	1797	2115	2392	3067	2598
	3–4	115	105	108	247	482	809

Each value is given for chosen example intervals of age and SES values taken from an overall continuous range, where the lower age/SES value of the interval is inclusive and the upper age/SES value is exclusive

### Model

**Fig. 1:**
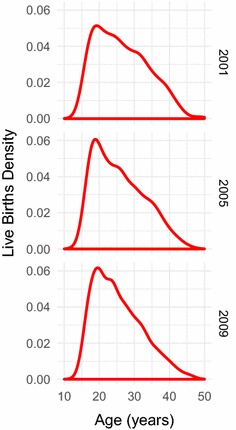
Kernel density estimate of live births over age. Non-parametric estimate of the distribution over age of women experiencing live births in the years 2001, 2005, and 2009 in the Agincourt health and socio-demographic surveillance system (HDSS) study area in rural South Africa. The distributions show the standard skewed hill-shaped age-pattern for fertility as found in most other work

**Fig. 2:**
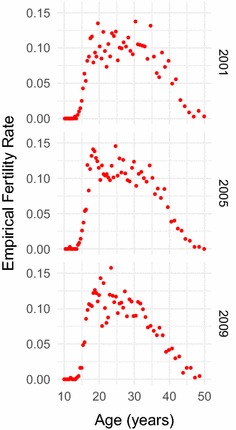
Empirical fertility rates over age. Empirical fractions of the number of women experiencing live births for each age centile, calculated for individuals living in the Agincourt health and socio-demographic surveillance system (HDSS) study area in rural South Africa in the years 2001, 2005, and 2009. The empirical probabilities show the standard skewed hill-shaped age-pattern for fertility as found in most other work

In order to obtain insight into what is happening within our dataset, we relied on regression methods where fertility rate acted as our dependent variable and covariates such as age, SES, and education acted as independent variables. Though there are no generally accepted nonlinear models of fertility over the other covariates, some have been described for fertility over age [17]. Indeed a definite hill shape skewed to lower ages can be seen in both the kernel density estimate of women experiencing live births over various years (Fig. 1) and in plots of the empirical fertility rates calculated for individuals grouped into age centiles (Fig. 2). In order to be certain that we captured this relationship, we used a parametric model for fertility over age and incorporated further covariates by allowing the parameters of our parametric model to be dependent on the other covariates. Various work has shown the age-pattern of fertility to contain a secondary earlier age peak credited to premarital fertility [6]. However as our data does not show significant evidence of this second peak (perhaps due to the nature of the binning we used) we therefore chose the Gamma distribution, a standard model for fertility over age, as our parametric form for our fertility rate for individual *i*, $p_i( a,{\mathbf{x}})$, over age *a* and dependent on further covariates ${\mathbf{x}}$, i.e.

1 $$\begin{aligned} p_i\left( a;{\mathbf{x}} \right)&= \text{Pr}\left( Y_i = 1 | a, {\mathbf{x}} \right) \nonumber \\&= \text{Gamma}\left( a | \alpha ({\mathbf{x}}) , \beta ({\mathbf{x}}) \right) \nonumber \\&= \frac{a^{\alpha ({\mathbf{x}}) - 1} e^{-a/\beta ({\mathbf{x}}) }}{\Gamma \left( \alpha ({\mathbf{x}}) \right) \beta ({\mathbf{x}}) ^{\alpha ({\mathbf{x}}) }} \end{aligned}$$

where the fertility indicator $Y_i$ of individual *i* is equal to 1 if the individual experienced a live birth for covariates *a* and ${\mathbf{x}}$ and equal to 0 otherwise, $\Gamma ( \cdot)$ is the gamma function, and $\alpha ({\mathbf{x}})$ and $\beta ({\mathbf{x}})$ are our shape and scale parameters which depend on our other covariates.

**Fig. 3:**
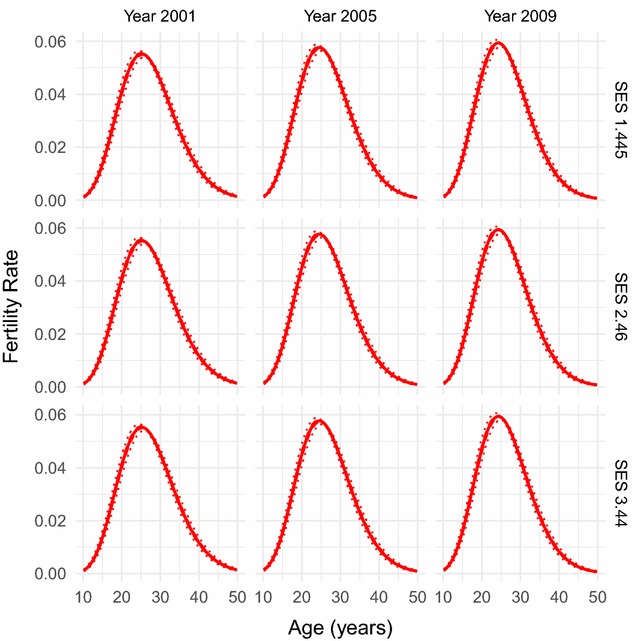
Fitted fertility rates over age. Fertility rate over age as fitted by our combined parametric and semi-parametric model, for socio-economic status values of 1.445, 2.46, and 3.44, and years 2001, 2005, and 2009. Parametrically bootstrapped confidence intervals (from 1000 samples of the model) are shown for the 50% level (dashed lines) and 95% level (dotted lines). The model has managed to capture the standard skewed hill-shape of the age-pattern as found in the raw data and in many fertility age-patterns in the literature

**Table 2:** Average fertility rates for different ranges of age (in years) and socio-economic status for each year, given to aid comparison of the analytical results to the data

Age	SES	Years					
		2001	2003	2005	2007	2009	2011
10–20	1–2	0.0501	0.0501	0.0608	0.0580	0.0724	0.0516
	2–3	0.0386	0.0352	0.0504	0.0472	0.0519	0.0408
	3–4	0.0355	0.0423	0.0349	0.0310	0.0497	0.0320
20–30	1–2	0.1006	0.1140	0.1296	0.1357	0.1269	0.1252
	2–3	0.0998	0.0959	0.1059	0.1060	0.1104	0.1120
	3–4	0.0645	0.0969	0.0777	0.0919	0.1057	0.1021
30–40	1–2	0.1000	0.0795	0.0918	0.0885	0.0846	0.1281
	2–3	0.0912	0.0754	0.0944	0.0756	0.0773	0.0936
	3–4	0.0798	0.0749	0.0912	0.0897	0.0814	0.0715
40–50	1–2	0.0355	0.0310	0.0206	0.0322	0.0273	0.0191
	2–3	0.0156	0.0242	0.0157	0.0179	0.0183	0.0207
	3–4	0.0077	0.0111	0.0077	0.0229	0.0223	0.0120

Each value is given for chosen example intervals of age and SES values taken from an overall continuous range, where the lower age/SES value of the interval is inclusive and the upper age/SES value is exclusive

For the functional forms of $\alpha ({\mathbf{x}})$ and $\beta ({\mathbf{x}})$, due to the lack of established models, we employed the method of Gaussian process regression for its flexibility and nonlinearity. A detailed description of Gaussian process regression can be found in [21]. In simple terms, Gaussian process regression is a method that aims to find a distribution over functions $f( \cdot)$ that relates a set of covariate observations $X = \left\{ {\mathbf{x}} _i\right\}$ to a set of dependent variable observations ${\mathbf{y}} = \left\{ y_i\right\}$ by $y_i = f\left( x_i\right) + \epsilon _i$ where $\epsilon _i$ is Gaussian noise. By incorporating our data we can calculate a posterior distribution of possible functions, where predictions of new function values ${\mathbf{f}} ^{*}$ for new observations with covariates $X^{*}$ can be drawn from the posterior predictive distribution

2 $${\mathbf{f}} ^{*} | X^{*} , X , {\mathbf{y}} \sim N\left( \pmb{\mu },\varvec{\Sigma } \right)$$

where

3 $$\pmb{\mu }= \varvec{K}\left( X^{*},X\right) \varvec{K}\left( X,X\right) ^{-1} {\mathbf{y}}$$

4 $$\varvec{\Sigma }= \varvec{K}\left( X^{*},X^{*}\right) - \varvec{K}\left( X^{*},X\right) \varvec{K}\left( X,X\right) ^{-1}\varvec{K}\left( X,X^{*}\right)$$

though the best prediction, and therefore the typically chosen function, comes from the mean of the distribution.

The method is called semi-parametric as we do not get a parametric relationship between the dependent and independent variables as a result, but instead parameters are used to define the covariance function. There are many different covariance functions to choose from, but a standard choice that we used in this analysis is the squared exponential covariance function, which results in a smooth and continuous relationship between our dependent and independent variables, and is defined as

5 $$\begin{aligned} \varvec{K}\left( {\mathbf{x}} _i,{\mathbf{x}} _j\right) = \sigma ^{2}_{f} \exp \left[ -\frac{1}{2}\left( {\mathbf{x}} _i - {\mathbf{x}} _j\right) ^{T} \varvec{M} \left( {\mathbf{x}} _i - {\mathbf{x}} _j\right) \right] + \sigma ^{2}_{n} \delta _{ij} \end{aligned}$$

where ${\mathbf{x}} _i$ is the covariate vector for observation *i*, $\delta _{ij}$ is the Kronecker delta which simply constrains that term to only appear when $i=j$, and the parameters of our covariance function are the noise variance $\sigma ^{2}_{n}$ accounting for the noise in the data, the signal variance $\sigma ^{2}_{f}$ which governs the size of the covariance between pairs of observations, and $\varvec{M} = \text{diag}\left( {\mathbf{l}} \right) ^{-2}$ where ${\mathbf{l}}$ is the vector of length parameters (one length parameter for each covariate). The length parameter for a particular covariate essentially governs how much our function varies over that covariate. For a small length parameter $f(x)$ would vary greatly over *x*, and for a large length parameter the relationship would essentially be flat. The values of these parameters (both the length parameters and the two variances) were found by using the maximum likelihood method as described in Rasmussen and Williams [21].

By fitting these parameters to the data, we allow the data to inform both the magnitude of the variance of $f(x)$ at each individual value of *x* and how far the covariance of $f(x)$ extends over *x*. Therefore these parameters essentially dictate both the magnitude of variation and frequency of fluctuations of $f(x)$ over *x*, without dictating a precise parametric form for $f(x)$. Though we could choose any function $f(x)$ as our estimate from the distribution $N\left( \pmb{\mu },\varvec{\Sigma } \right)$, the mean minimises the expected squared error between our outputs *y* and our estimates $f(x)$ and therefore gives the best result.

One way to think of this method of combining Gaussian process regression with parametric regression is that the Gaussian process regression smooths over the other covariates the parameters of our model for the role of age. Fitting Gaussian distributions of functions to the estimates of $\alpha ({\mathbf{x}})$ and $\beta ({\mathbf{x}})$ found from parametrically fitting over age allows the data to both give an initial noisy estimate of the functional forms of $\alpha ({\mathbf{x}})$ and $\beta ({\mathbf{x}})$ and then to smooth over them by defining, given these initial estimates, the magnitude and frequency of how $\alpha ({\mathbf{x}})$ and $\beta ({\mathbf{x}})$ vary over ${\mathbf{x}}$.

We guarded against overfitting of the Gaussian process through use of a smoothing prior for the length parameter for SES, a gamma distribution with shape parameter 6 and scale parameter 0.25. Wider and thinner priors were also tried to see what effect the prior choice had on the results, but little to no differences were found.

In order to find which combination of covariates would be best to include in the model, as well as to decide on how many quantiles should be used for binning as described in the previous section, a combination of cross validation and goodness-of-fit tests were used. To measure the predictive performance of each possible model choice tenfold cross validation was used, where the performance was measured by their Briers score [22]

6 $$S_B = \frac{1}{N} \sum ^{N}_{i=1} \left( Y_i - p_i\left( a,{\mathbf{x}} \right) \right)^2$$

which compares the fitted probability of fertility to the actual fertility status of each of the *N* observations. Due to the treatment of the problem as a regression, and therefore having to bin the data, goodness-of-fit tests to the unbinned data could not be performed. Instead we performed Kolmogorov–Smirnov (KS) tests comparing the fitted marginal fertility probabilities over age to the empirical marginal fertility rates over age [23]. Model choices were rejected at a 5% significance level, where the Bonferonni method was used to mitigate against the possibility of rejecting by chance due to performing a large number of tests [24]. It was found that, though it was possible to include education in addition, it was best to simply focus on age and SES. This is down to two reasons. First, there are more missing values for the Agincourt HDSS education data than the SES data. Second, that introducing more covariates leads to worse fits when using maximum likelihood on the parametric model over age.

## Results

**Fig. 4:**
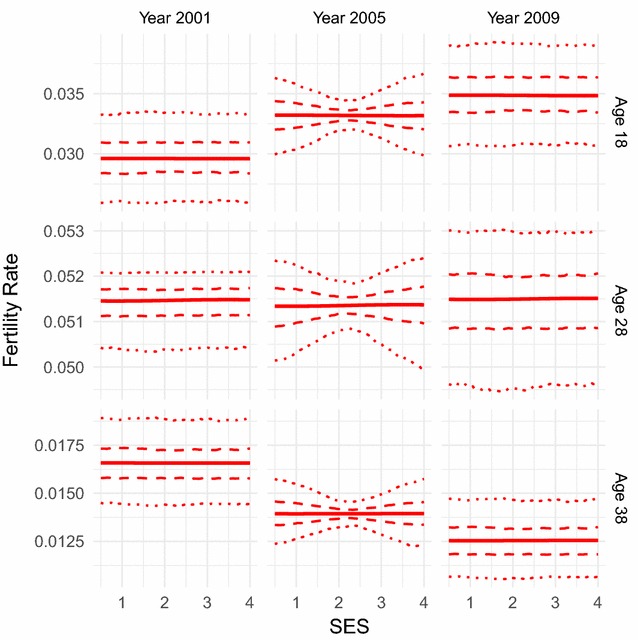
Fitted fertility rates over socio-economic status. Fertility rate over socio-economic status (SES) as fitted by our combined parametric and semi-parametric model, for age values of 18, 28, and 38, and years 2001, 2005, and 2009. Parametrically bootstrapped confidence intervals (from 1000 samples of the model) are shown for the 50% level (dashed lines) and 95% level (dotted lines). The model manages to capture various details of the fertility-SES pattern, such the time trends of fertility for the different age groups

The resulting fitted forms for fertility rate over age and SES for a range of years between 2001 and 2011 can be seen in Figs. 3 and 4. Figure 3 shows how fertility rate varies over age. We can see that the model has captured the standard skewed relationship, as we would expect from our choice of parametric model. Fertility increases rapidly from mid-adolescence to peak in mid- to late-20s, before more gently decreasing until early-50s. We observed little age-pattern fertility changes for different SES values. However there is a slight increase in peak fertility over age as time increases, which goes against the fertility decline which has been observed elsewhere [2]. This increase, though, is small and so probably does not necessitate much explanation. However it is consistent with an increase in peak fertility we see in the raw data (Figs. 1, 2).

The relationship between fertility rate and SES can be seen in Fig. 4 for a variety of ages and years. It must be first noted that fertility rate varies little over SES for any year or age, which would be consistent with how homogeneous we know the individuals in the study area to be. Overall it would appear to be almost constant over SES for the individual age-year combinations. The fertility-SES relationship varies over time but differently for different ages, where the plots suggest that adolescent pregnancy has actually increased over time and late-30s pregnancy has actually decreased over time. Late-20s pregnancy, which is closer to the peak in the fertility age-pattern, has a very small magnitude of variation over time with no consistent trend.

**Fig. 5:**
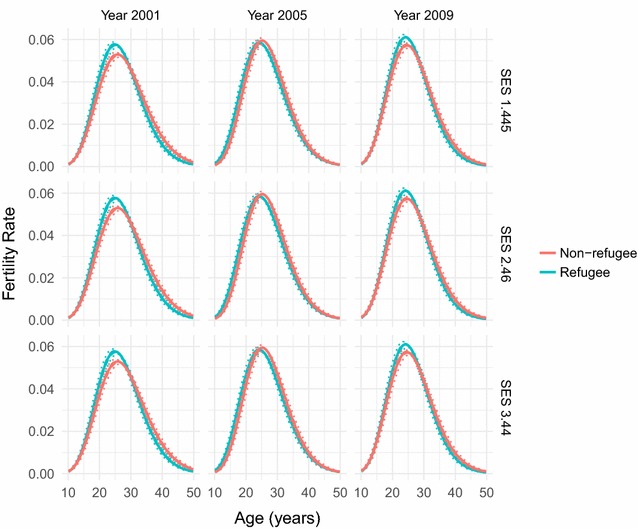
Fitted fertility rates over age for refugees and non-refugees. Fertility rate over age as fitted by our combined parametric and semi-parametric model, for socio-economic status values of 1.445, 2.46, and 3.44, and years 2001, 2005, and 2009, for the refugee and non-refugee populations of Agincourt. Parametrically bootstrapped confidence intervals (from 1000 samples of the model) are shown for the 50% level (dashed lines) and 95% level (dotted lines). These fertility age-patterns only show slight differences between the populations and the overall population, reflecting the convergence of fertility between them

**Fig. 6:**
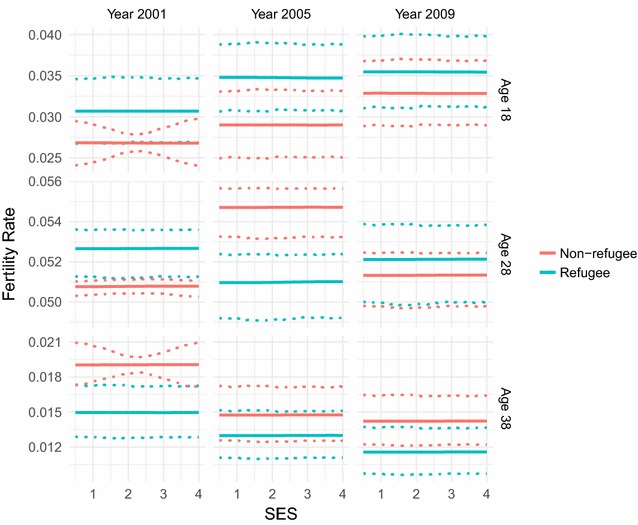
Fitted fertility rates over socio-economic status for refugees and non-refugees. Fertility rate over socio-economic status (SES) as fitted by our combined parametric and semi-parametric model, for age values of 18, 28, and 38, and years 2001, 2005, and 2009, for the refugee and non-refugee populations of Agincourt. Parametrically bootstrapped confidence intervals (from 1000 samples of the model) are shown for the 95% level (dotted lines). Some slight differences between the refugee and non-refugee populations are shown, particularly in variation of fertility over time for 28 years old individuals. However these differences are slight, reflecting the convergence of fertility between the two populations

We also performed the same analysis (using the same covariates and quantiles) on the individual refugee and non-refugee populations, achieved by splitting the dataset into refugee and non-refugee sub-datasets according to whether the individuals were marked out as refugees in the dataset or not. Significant differences have been shown in fertility levels between the Mozambican refugee population who came over to the study area in response to the civil war, and the South African population, though it has been shown that the populations have converged in recent years [8]. The results here (Figs. 5, 6) do show some differences, though indeed the fertility patterns of each population appear to have become quite similar. The non-refugees show the same increase in peak fertility as the overall population, whilst the refugees have lower fertility at both earlier and later years. The fertility patterns over SES remain constant, and for individuals in their late teens and late-30s we see the same patterns over time as we have before. However there is more variation over time for individuals in their late-20s for both populations, though in opposite directions, with refugee fertility increasing substantially in 2005 before settling back down again by 2009 and non-refugee fertility varying in the opposite direction before coming back as well. It should be noted that there is a severe overlap in the confidence intervals of the fertility SES-patterns for most ages and years. Overall this implies that there is not a great deal of variation from the overall population when differentiating by refugee status, consistent with the convergence of fertility in the refugee and non-refugee populations.

The combination of results does imply a linear trend of fertility over SES for this population, but this does not dispute the usefulness of incorporating further covariates other than age by Gaussian process regression as this still overcomes the need to make unfounded a priori assumptions of linearity.

## Discussion

By combining a parametric regression of fertility rate over age with the use of Gaussian process regression to bring in further covariates such as SES, we produce an improvement in robustness to the modelling of fertility. The parametric part of our model successfully captures the well known skewed hill relationship between fertility and age that can be seen both in empirical plots of our own data shown in Figs. 1 and 2, as well as in many other research papers that have used empirical calculations or other or similar parametric models to model the fertility age-patterns of sub-Saharan Africa [3, 6, 10, 11, 14, 15, 17].

The semi-parametric part of our model, using Gaussian process regression over other covariates, successfully manages to model the SES pattern of fertility without simply assuming the relationship to be linear as other work has done [2, 3, 16]. This gives the potential to capture more detail within the relationship and provide greater insight to what has been happening to fertility in the Agincourt study area between 2001 and 2011. We found how the magnitude of variation of fertility over SES is quite small, suggesting that SES does not have as big an impact on fertility as we would think, reflecting the homogeneity of the population built into the model by the smoothing prior. In fact it would appear to be almost constant, and certainly quite linear. Though this means that a generalised linear model could have been used in this case, the incorporation of this modelling technique into fertility modelling is still useful. We had no a priori justification for a linear model, and using one would definitely have restricted our results such that we would have no chance of capturing possible nonlinearities. It is also bad practice to justify heavy assumptions a posteriori on the relationships we are modelling. For other similar modelling problems where linear models are commonly used, and for further fertility modelling itself in other types of populations, this method allows for much more relaxed assumptions about relationships where we have no a priori justification for stricter assumptions. Otherwise, our results have also shown that adolescent fertility does appear to have increased over time, whilst later life fertility appears to have decreased. Overall, the flexibility and nonlinearity of the method allows for the potential capture of much more information than a single linear coefficient can show, and therefore increases the robustness of the results.

An interesting detail is that the peak of the fertility age-pattern found by our model appears to increase over time, which, though the variation is small, contradicts work done on fertility trends over time that have found a significant decline in fertility over the past several decades [2, 9, 11, 14, 25]. This could simply be a quirk of the data, or could be due to some deeper phenomenon happening in the study area in recent years.

When differentiating by refugee status, some differences are seen between refugee and non-refugee populations. However these differences are not so substantial to mark out the populations as significantly different from the overall population, perhaps due to the convergence in fertility of the two populations shown in the literature.

There are limitations in the work presented here. Though a lot of effort is undergone by the Agincourt research unit to ensure the reliability of the HDSS data, as detailed elsewhere [18], there are some errors, misreporting, and missing data that we are unable to account for. The dataset is of a size and quality though that these only produce minimal issues and do not seriously undermine the results presented here [26]. The method itself also comes with some limitations, principally produced by the use of a parametric model and the decision to use regression techniques. The parametric regression must be performed for each combination of values for the non-age covariates, which means that introducing further covariates reduces the performance of the regression, a situation that can only be mitigated by using more data or not relying on a parametric model. However the use of the parametric model allows us to definitely capture the age-pattern shown in our empirical data and in fertility age-patterns for many populations in the literature (though admittedly prevents us from being able to capture possible details such as a second fertility peak). In order to use regression techniques we have to bin the observations in the data to quantiles, which results in the removal of information. We mitigated against this by using cross validation and goodness-of-fit techniques to choose between different numbers of quantiles to use. The use of regression techniques also ensures we can produce visualisations of the relationships that can give us insight in to what is going on, and not just predictions alone.

Further research to extend our method might include the following. First, to overcome the limitations mentioned, Gaussian process regression could be used for age rather than relying on a parametric model. A probabilistic classification technique such as Gaussian process classification could be used instead of regression techniques to overcome the issue of having to bin the observations together. In order to examine the apparent lack of a fertility decline in our results, and to make the research into the fertility decline more robust, nonlinear modelling techniques such as Gaussian process regression could be applied to the fertility time series of the study area. Also, other outcomes than fertility, that have also been analysed using less innovative methods, could be explored with this same technique. Finally, as mentioned before, a second fertility peak has been found by previous studies to exist in the Agincourt population, which our model is restricted from capturing . It would also be of interest to see what happens when the parametric fertility-age model used here is replaced with a double peaked model such as that proposed by Peristera and Kostaki [17].

## Conclusion

Though the measurement of patterns of fertility over different covariates is of great importance to the analysis of population dynamics, most research still relies on methods such as empirical calculations and linear models to do so which are open to issues such as susceptibility to noise and assuming a linear relationship without justification. Here we have presented a method to incorporate further covariates into a nonlinear parametric model of fertility over age by regressing the parameters of the model on these covariates using Gaussian process regression, which is both nonlinear and flexible. This allows us to limit our assumptions of the relationships between fertility and these covariates to simply being smooth and continuous. We successfully applied the model to data from the Agincourt health and socio-demographic surveillance system collected between 2001 and 2011, an annual census update collecting demographic (births, deaths, and migration) and health data on a poor rural region of South Africa, to examine how fertility varies over age and socio-economic status (SES). Our method managed to capture the expected age-pattern of fertility, and gave further insights into how fertility varies over SES in the Agincourt study area. The magnitude of the fertility variation over SES is small, essentially constant, reflecting the homogeneity of the study area population. This linearity produced by the Gaussian process regression however does not undermine the use of the method as there is no a priori reason to assume the relationship to be linear, and by relaxing the initial assumptions our model makes we have therefore substantially increased the robustness of these results. The results also show that the fertility age-pattern peak appears to increase over time, which is inconsistent with a lot of established work on the well known fertility decline in sub-Saharan Africa. Further work should therefore apply nonlinear methods to the fertility time series in the area to examine whether this is a local phenomenon or simply that the trend in the peak is not reflected in fertility as a whole. A less restrictive form for the fertility age-pattern should also be incorporated into the model to see if the second fertility peak found in the literature can be captured.

## Abbreviations

HDSS: health and socio-demographic surveillance system
SES: socio-economic status

## References

[1] Houle B, Clark SJ, Gómez-Olivé FX, Kahn K, Tollman SM (2014). The unfolding counter-transition in rural South Africa: mortality and cause of death, 1994–2009. PLoS ONE.

[2] Burger RP, Burger R, Rossouw L (2012). The fertility transition in South Africa: a retrospective panel data analysis. Dev South Afr.

[3] Camlin CS, Garenne M, Moultrie TA (2004). Fertility trend and pattern in a rural area of South Africa in the context of HIV/AIDS. Afr J Reprod Health.

[4] Arthur S, Bangha M, Sankoh O (2013). Review of contributions from HDSSs to research in sexual and reproductive health in low-and middle-income countries. Trop Med Int Health.

[5] Kravdal Ø (2002). Education and fertility in sub-saharan africa: Individual and community effects. Demography.

[6] Garenne M, Tollman S, Kahn K (2000). Premarital fertility in rural South Africa: a challenge to existing population policy. Stud Fam Plann.

[7] Palamuleni M, Adebowale A (2014). Patterns of premarital childbearing among unmarried female youths in sub-Saharan Africa: evidence from demographic health survey. Sci. Res. Essays.

[8] Williams J, Ibisomi L, Sartorius B, Kahn K, Collinson M, Tollman S, Garenne M (2013). Convergence in fertility of South Africans and Mozambicans in rural South Africa, 1993–2009. Glob Health Action.

[9] Moultrie TA, Timæus IM (2003). The South African fertility decline: evidence from two censuses and a demographic and health survey. Popul Stud.

[10] Garenne ML, Tollman SM, Collinson MA, Kahn K (2007). Fertility trends and net reproduction in Agincourt, rural South Africa, 1992–2004 1. Scand J Public Health.

[11] Kirk D, Pillet B (1998). Fertility levels, trends, and differentials in sub-Saharan Africa in the 1980s and 1990s. Stud Fam Plann.

[12] Palamuleni M, Kalule-Sabiti I, Makiwane M, Amoateng AY, Heaton TB (2007). Fertility and childbearing in South Africa. Families and households in post-apartheid South Africa: socio-demographic perspectives.

[13] Nilses C, Lindmark G, Munjanja S, Nyström L (1997). Trends in fertility patterns of women in rural Zimbabwe. Health Care Women Int.

[14] Moultrie TA, Timaeus IM (2002). Trends in South African fertility between 1970 and 1998.

[15] Garenne M, Zwang J (2006). Premarital fertility in Namibia: trends, factors and consequences. J Biosoc Sci.

[16] Ayele DG (2015). Determinants of fertility in Ethiopia. Afr Health Sci.

[17] Peristera P, Kostaki A (2007). Modeling fertility in modern populations. Demogr Res.

[18] Kahn K, Collinson MA, Gómez-Olivé FX, Mokoena O, Twine R, Mee P, Afolabi SA, Clark BD, Kabudula CW, Khosa A (2012). Profile: Agincourt health and socio-demographic surveillance system. Int J Epidemiol.

[19] Kahn K, Tollman SM, Collinson MA, Clark SJ, Twine R, Clark BD, Shabangu M, Gómez-Olivé FX, Mokoena O, Garenne ML (2007). Research into health, population and social transitions in rural South Africa: data and methods of the Agincourt Health and Demographic Surveillance System1. Scand J Public Health.

[20] Collinson MA, Clark SJ, Gerritsen AM, Byass P, Kahn K, Tollman S. The dynamics of poverty and migration in a rural South African community, 2001–2005. CSSS Working Paper Series, no. 92. 2009. p. 1–38.

[21] Williams CK, Rasmussen CE (2005). Gaussian processes for machine learning.

[22] Brier GW (1950). Verification of forecasts expressed in terms of probability. Mon Weather Rev.

[23] Wasserman L (2013). All of statistics: a concise course in statistical inference.

[24] Bland JM, Altman DG (1995). Multiple significance tests: the Bonferroni method. BMJ.

[25] Garenne M, Joseph V (2002). The timing of the fertility transition in sub-Saharan Africa. World Dev.

[26] Fottrell E, Byass P, Berhane Y (2008). Demonstrating the robustness of population surveillance data: implications of error rates on demographic and mortality estimates. BMC Med Res Methodol.

